# Thinking While Playing: Football-Specific Dual-Task Performance and Coach-Rated Talent in Youth Football

**DOI:** 10.3390/sports14090399

**Published:** 2026-09-10

**Authors:** Juan Miguel Ramírez Lucas, Juan Antonio Párraga Montilla, Manuel Herrero Sánchez, José Carlos Cabrera-Linares, Pedro Ángel Latorre Román

**Affiliations:** Department of Didactic of Music, Plastic and Corporal Expression, University of Jaén, 23071 Jaén, Spain; jmrl0014@red.ujaen.es (J.M.R.L.); jparraga@ujaen.es (J.A.P.M.); mherrsan@gmail.com (M.H.S.); platorre@ujaen.es (P.Á.L.R.)

**Keywords:** dual-task, youth football, talent identification, cognitive-motor performance, ecological validity

## Abstract

**Background:** Football performance emerges from the interaction of technical, physical, and perceptual-cognitive abilities, yet talent identification protocols often assess these components separately. This study examined whether football-specific dual-task (DT) performance, assessed using the Soccer Skills and Cognitive Aptitude Test (SoSCAT), could discriminate between coach-rated talent levels in youth football players. **Methods:** A total of 181 male football players aged 12–18 years participated in this cross-sectional study and were classified into U14 (n = 70), U16 (n = 63), and U18 (n = 48) categories. Based on coaches’ evaluations, players were also classified as more talented (n = 46) or less talented (n = 135). Participants completed football-specific technical, physical, cognitive, and DT assessments. **Results:** Coach-rated talented players demonstrated superior football-specific DT performance, with faster completion times under DT conditions (partial η^2^ = 0.190), a smaller increase in completion time (ΔTime; partial η^2^ = 0.095), lower DTC (r_r__β_ = −0.351), and fewer decision errors (r_r__β_ = −0.211). Older players showed better absolute DT performance and fewer decision errors, although ΔTime and DTC did not differ between age categories. Age, coach-rated talent status, league goals, and football-specific technical performance remained independently associated with SoSCAT DT performance (adjusted R^2^ = 0.500). ROC analysis showed good discriminative ability for SoSCAT DT performance (AUC = 0.822). **Conclusions:** Football-specific DT performance demonstrated good discriminative ability between coach-rated talent levels. The SoSCAT may represent a valuable complementary tool for multidimensional player assessment by integrating football-specific technical execution and perceptual-cognitive demands. However, its interpretation should consider the cross-sectional design, absence of biological maturation assessment, and reliance on coach-rated talent classification.

## 1. Introduction

Football is one of the most widely practised sports across the world and plays a particularly influential role during childhood and adolescence. It is classified as an invasion and open-skill sport, in which players must constantly adapt their behaviour in response to unpredictable and rapidly changing stimuli from both teammates and opponents [1]. Successful performance in such environments relies not only on physical attributes such as speed, including acceleration, deceleration and rapid changes in direction, but also on technical proficiency in actions involving ball mastery, such as control, dribbling and short passing [2]. Beyond these aspects, football performance is strongly dependent on the continuous interaction between perception and action in dynamic and time-constrained situations [3].

Identifying sporting talent during adolescence remains one of the greatest challenges in football development programmes [4]. Although physical and technical characteristics have traditionally been considered the main criteria for player selection, growing evidence indicates that successful performance also depends on perceptual-cognitive abilities, such as attention, anticipation and decision-making under pressure [5]. Consequently, contemporary talent identification models increasingly advocate multidimensional approaches capable of integrating physical, technical and cognitive components to better capture the complexity of football performance [4,6].

A crucial element in this interaction is the player’s ability to divide attention among multiple cues in the playing environment, such as the ball, the positioning of opponents and the movement of teammates in order to make rapid and effective decisions [7]. Visual search behaviour and the anticipation of situational probabilities have been identified as fundamental cognitive processes underpinning football performance [8]. Elite players, in particular, are frequently exposed to intense visuomotor demands in which decisions must be made within milliseconds. The ability to process visual information efficiently is therefore recognised as a decisive factor for success in fast-paced sports, where saccadic eye movements provide a crucial mechanism for coordinating visuomotor responses and anticipating future actions [9].

Empirical evidence has shown that athletes with higher expertise consistently outperform their less-skilled peers in perceptual, cognitive and motor tasks, largely due to faster visuomotor reaction times [10]. Expert players also demonstrate superior visual capacities and adopt more effective visual search strategies, enabling them to focus on task-relevant information and to suppress irrelevant stimuli. These perceptual-cognitive advantages not only enhance on-field decision-making but also contribute to the acquisition and refinement of motor skills [11,12].

Within this framework, football performance requires players to simultaneously process multiple sources of information while executing technical actions. For instance, they must attend to a teammate’s request, anticipate the trajectory of the ball and adjust their positioning relative to an opponent, while also coping with environmental factors such as crowd noise or instructions from the coach [8,13]. This close interdependence between executive mechanisms responsible for action and the perceptual-cognitive processes guiding decision-making is therefore central to understanding performance in football [14].

Psychomotor and perceptual-cognitive abilities are particularly relevant during youth development, as successful performance in football requires the coordinated integration of perceptual information, cognitive processing, and motor responses. Abilities such as psychomotor speed, visuospatial processing, working memory, planning, and motor control may contribute to players’ capacity to rapidly process environmental information and adapt their actions within dynamic and unpredictable game situations [15,16]. In this context, the Vismem-Plan and Wom-Rest tests were selected to assess complementary cognitive processes, including working memory, visuospatial processing, processing speed, and planning, thereby providing measures of cognitive performance potentially relevant to football-specific decision-making and cognitive-motor integration [17,18].

The dual-task (DT) paradigm offers a valuable framework for investigating this interaction between cognition and action. It refers to the concurrent execution of a motor task and a cognitive task, which requires the allocation of attentional resources across both domains [19,20]. According to Plummer and Eskes [21] attention is inherently limited, so the simultaneous performance of two tasks often leads to performance decrements in one or both. This phenomenon, influenced by factors such as cognitive capacity and motor development [22], is described as the dual-task cost (DTC) [23], and can be quantified using the formula proposed by Plummer and Eskes [21].

Research consistently shows that higher-level athletes display lower DTC values than their less-skilled counterparts, which indicates a greater ability to preserve performance under multitasking conditions [24,25]. In football, this suggests that players who are more efficient in DT exercises are able to perform technical actions with higher precision and at the right moment [7]. However, several studies have also reported that the inclusion of cognitively demanding elements produces performance decrements, with visual tasks in particular creating greater interference than auditory ones due to the overlap in spatial processing requirements [26,27].

The observed differences between expert and novice athletes further support the use of DTC as a criterion for distinguishing levels of expertise in various sports [28]. In football, there is ongoing debate as to whether players at higher levels excel not only in traditional physical and technical tasks but also in executive and cognitive abilities. This line of inquiry provides an important complement to standardised cognitive assessments and sport-specific performance tests [28,29].

Adolescence represents a particularly relevant period for talent identification because players experience substantial physical, technical and cognitive development while simultaneously being exposed to increasingly demanding competitive environments [30,31,32]. During these years, differences in biological maturation, accumulated practice and perceptual-cognitive development may strongly influence performance and selection decisions. Therefore, assessment tools capable of distinguishing players beyond isolated physical or technical qualities are especially valuable during this developmental stage [4,6].

Within this multidimensional framework of talent identification, football-specific technical skills [33], together with perceptual-cognitive capacities [29], represent crucial components in evaluating player potential. However, most talent identification protocols continue to assess technical, physical, and cognitive domains separately, limiting their ability to capture the interaction between technical execution and cognitive processing under representative performance conditions. In this context, the Soccer Skills and Cognitive Aptitude Test (SoSCAT) provides a football-specific approach for assessing technical performance while simultaneously introducing cognitive interference, enabling performance to be evaluated under both single and DT conditions [34]. Such an approach may provide additional information for multidimensional player assessment by identifying differences in players’ ability to maintain effective football-specific performance under cognitively demanding conditions.

Consequently, the aim of this study was to examine whether football-specific performance under DT conditions, particularly completion time under interference, completion time increase (ΔTime), and DTC, could discriminate between coach-rated talent levels in youth football players. We hypothesised that players classified by their coaches as more talented would demonstrate superior performance under DT conditions, characterised by shorter completion times, a smaller increase in completion time (ΔTime), lower DTC, and fewer decision errors. Furthermore, we expected the SoSCAT to demonstrate good discriminative ability between coach-rated talent groups, owing to its capacity to simultaneously assess football-specific technical execution and perceptual-cognitive performance within an ecologically valid context.

## 2. Materials and Methods

### 2.1. Participants

A cross-sectional design was applied using a convenience sample, involving a total of 181 healthy male football players aged 12–18 years (mean age: 14.75 ± 1.81 years). Participants were recruited from nine teams across three semi-professional clubs located in southern Spain. All players competed actively in official regional leagues, adhering to a consistent routine of three weekly training sessions (approximately 90 min each) and one competitive match on weekends. Eligibility criteria included possession of a valid federation license and the absence of injuries that could interfere with football performance during the three months prior to testing. Written informed consent was obtained from the participants’ parents or legal guardians before participation. The study adhered to the principles of the Declaration of Helsinki (2013) and received approval from the Ethics Committee of the University of Jaén (Ref. 20231218/ENE.TES).

The sample included players competing in three official regional leagues of the Andalusian Royal Football Federation (3rd division: n = 66; 2nd division: n = 96; 1st division: n = 19). In line with federation criteria, players were classified into three age categories: under-14 (U14, n = 70), under-16 (U16, n = 63) and under-18 (U18, n = 48). Furthermore, players were dichotomously classified as more talented (n = 46) or less talented (n = 135) according to the judgement of their respective head coaches. In total, nine coaches participated in this classification process, with each of the nine teams being evaluated by its respective head coach. All coaches held at least a UEFA C (Level 1 equivalent) coaching qualification and were officially licensed by both the Royal Spanish Football Federation and the Andalusian Football Federation. Each coach classified only the players from his own team based on continuous observations accumulated throughout the competitive season, considering their overall performance during training sessions and official matches. All nine coaches received the same instructions and were asked to select between four and six players from their respective teams (typically comprising 19–22 players) whom they considered to demonstrate superior overall football performance relative to their teammates. No predefined technical, tactical, physical, or cognitive criteria or standardized rating scale were imposed; rather, coaches were asked to provide an overall judgement based on their professional experience and accumulated observations during training and competition [5]. The final classification was determined by the respective head coach and subsequently provided to the research team in written form. Each player was evaluated only by his own head coach; therefore, no consensus procedure across coaches or assessment of inter-rater reliability was performed.

### 2.2. Materials and Testing

#### 2.2.1. Anthropometrics and Sociodemographic Measures

Body mass was measured using a weighing scale (Seca 899, Hamburg, Germany), and body height was measured with a stadiometer (Seca 222, Hamburg, Germany). The participants’ body mass index (BMI) was calculated by dividing their body mass (in kilograms) by the square of their body height (in metres). Football-specific characteristics were recorded, including experience in federated football (in years), category, number of goals scored during the season (in league matches), and usual position on the pitch. Biological maturation status was not assessed in the present study; therefore, no maturity-related indicators, such as maturity offset or estimated age at peak height velocity (APHV), were available for analysis.

#### 2.2.2. Soccer-Skills Test

Technical skills were assessed using a dribbling test and a passing test that have been previously described and validated in youth football populations [35]. The combined performance in both tests was used to calculate the composite variable ∑Skill (passing + dribbling) [35]. Both tests were performed with official balls and test times were obtained using a stopwatch to the nearest 0.01 s [34]. These tests have demonstrated acceptable test–retest reliability (dribbling: r = 0.82; passing: r = 0.81; *p* < 0.001), supporting their use as reliable measures of football-specific technical performance under controlled conditions [35].

#### 2.2.3. Cognitive Test

Cognitive functions were assessed using the Vismem-Plan test, derived from Corsi’s block-tapping task [36] and the Wom-Rest recognition test, based on the classic symbol-search tasks of the WAIS [37]. Together, these instruments provide an integrated assessment of key processes such as working memory, spatial perception, processing speed, and planning ability, which were considered relevant to the continuous processing and updating of spatial information and rapid adaptation of motor responses required in football. Their digital application and psychometric properties have been previously documented in youth football players [34], supporting their use in the present study. Wom-Rest and Vismem-Plan data were available for a subsample of 136 participants because different cognitive assessment protocols were administered across participants.

#### 2.2.4. Physical Test

##### Countermovement Jump (CMJ)

Jump height was estimated from flight time using an infrared platform (Optogait, Microgate, Bolzano, Italy) [38]. During the CMJ, the subject was instructed to rest his hands on his hips while performing a downward movement to reach about 90° of knee flexion, followed by a maximal vertical jump [39].

##### 10-Metre Sprint Test

The running speed was calculated from the time (in seconds) measured using two double light barriers (WITTY photocell; Microgate Srl, Bolzano, Italy; accuracy 0.001 s), which were placed at the beginning and the end of a corridor [40].

##### 505 Change-of-Direction Test (505 COD)

The test was performed by sprinting for 10 m and then pivoting and sprinting back to the starting line [41]. Time was measured using a WITTY photocell. The mean performance for the 505 COD was calculated from the following formula: 505 COD average = (Time 505 COD with right leg + Time 505 COD with left leg)/2 [42]. Additionally, a change-of-direction deficit was derived relative to the 10 m sprint time to isolate change-of-direction ability from linear speed [41,42].

#### 2.2.5. Dual-Task Assessment

Cognitive interference and football-specific DT performance were evaluated using the Stroop test and the SoSCAT, respectively.

##### Stroop Test

The Stroop test [43,44] was employed to evaluate inhibitory control and attentional processes by requiring participants to read or name words and colours under congruent and incongruent conditions, following the procedure fully described in [34].

##### Soccer Skills and Cognitive Aptitude Test (SoSCAT)

The SoSCAT, recently validated in youth football populations by Latorre Román et al. [34], was designed to integrate football-specific technical actions including ball driving, dribbling, short passing, and ball control within a circuit that replicates game-like demands. To increase cognitive demands, the test was conducted under two different conditions (i.e., under conditions with and without visual stimuli) using visual stimuli presented through the WITTY system, which required players to adapt their actions in real time according to the displayed signals. The test simultaneously records execution time and decision accuracy (i.e., correct or incorrect route selection). In addition, the increase in completion time from the single-task to the DT condition (ΔTime) and the proportional DTC were calculated to quantify the performance decrement associated with the introduction of cognitive interference. DTC (%) = [(DT completion time-single-task completion time)/single-task completion time] × 100. By combining football-specific motor execution with decision-making under temporal pressure, the SoSCAT offers an ecologically valid approach to evaluating football performance and has demonstrated adequate reliability and validity parameters in youth football players [34].

**Figure 1:**
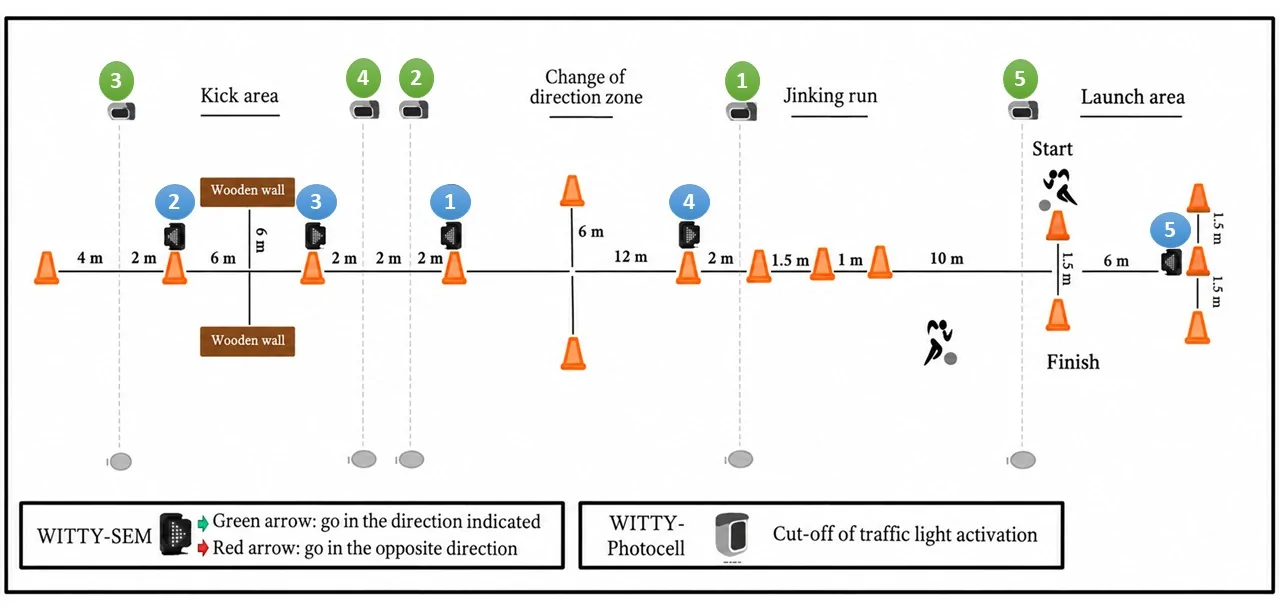
Soccer Skills and Cognitive Aptitude Test.

In Figure 1, blue numbers identify the WITTY-SEM devices that indicate the direction of movement, whereas green numbers identify the WITTY-Photocells. Each WITTY-Photocell is paired with the WITTY-SEM bearing the same number. When the participant crosses a WITTY-Photocell, the corresponding WITTY-SEM is activated and indicates the direction of movement. 

### 2.3. Procedure

All assessments were conducted during the competitive season. Testing sessions were scheduled on Tuesday, Wednesday, and Friday, to minimise the influence of match-related fatigue. Participants were instructed to refrain from vigorous physical activity during the 24 h preceding each testing session. All field-based assessments were carried out in the participants’ usual training environment (i.e., on an artificial grass football pitch). Three separate days were used to complete the entire evaluation protocol.

On the first day, the sociodemographic questionnaire was administered, and body weight and height were recorded. On the second day, following a standardised 10 min warm-up consisting of light aerobic running, changes in direction, and progressive sprints, participants received standardised instructions before testing. Participants completed two familiarisation trials before each performance test. No feedback was provided to the participants between recorded attempts. The order of the tests after the warm-up was as follows: (1) CMJ; (2) 10 m sprint test; (3) 505 COD; (4) football dribbling test; (5) football passing test; and (6) SoSCAT test. Participants were encouraged to perform maximally during all physical and football-specific tests. In the SoSCAT, the order of the single-task and DT conditions was randomised across participants, so that some players completed the single-task condition first, whereas others began with the DT condition. The best performance from the two recorded attempts was retained for subsequent analyses. In all trials, players rested for five minutes between attempts to minimise the influence of accumulated fatigue and ensure full recovery. On the third day, the cognitive tests (Vismem-Plan and Wom-Rest), together with the Stroop test used to assess cognitive interference, were administered individually under standardised conditions in a quiet room. All assessments were administered by the same trained research team following identical testing procedures.

### 2.4. Statistical Analysis

Data were analysed using SPSS (version 22.0; SPSS Inc., Chicago, IL, USA). Prior to the analyses, data normality and homogeneity of variance were assessed using the Kolmogorov–Smirnov and Levene’s tests, respectively. Descriptive statistics are presented as means ± standard deviations (SDs) or percentages (%), as appropriate. Differences across age categories (U14, U16, and U18) were examined using one-way analysis of variance (ANOVA), followed by Bonferroni post hoc tests when appropriate. For variables that violated the assumptions of normality, the Kruskal–Wallis test followed by Mann–Whitney U post hoc comparisons was used.

Comparisons between coach-rated talent groups (more talented vs. less talented) were performed using parametric or non-parametric methods, as appropriate. These comparisons were adjusted for age when appropriate, using separate analyses of covariance (ANCOVAs), with coach-rated talent status as the fixed factor, the corresponding performance variable as the dependent variable, and age as the covariate. Specifically, age-adjusted ANCOVAs were conducted for the 10-metre sprint test, football dribbling test, football passing test, ∑skill, Wom-Rest completion time, Stroop test interference, and SoSCAT single-task completion time. SoSCAT performance under DT conditions and ΔTime were adjusted for decision errors using separate ANCOVAs, with the corresponding SoSCAT measure as the dependent variable and decision errors as the covariate. Likewise, comparisons of SoSCAT DT completion time and ΔTime across age categories were performed using ANCOVA, with age category as the fixed factor and decision errors as the covariate. DTC was analysed using non-parametric methods because it did not meet the assumptions required for parametric analysis.

Effect sizes were calculated and reported with 95% confidence intervals (CIs). Eta squared (η^2^) was used for parametric comparisons across age categories and playing positions, epsilon squared (ε^2^) for non-parametric comparisons, Hedges’ g for unadjusted parametric comparisons between coach-rated talent groups, rank-biserial correlation (r_rb) for non-parametric comparisons between talent groups, and partial eta squared (partial η^2^) for adjusted comparisons. Pearson correlation analyses were conducted to examine the associations between SoSCAT performance and demographic, physical, technical, and cognitive variables, with correlation coefficients (r) reported alongside their 95% CI. Analyses involving Wom-Rest and Vismem-Plan were based on available cases (n = 136).

A multivariable linear regression analysis was subsequently performed to examine factors independently associated with SoSCAT completion time under DT conditions. Age, coach-rated talent status, number of league goals, 10-metre sprint performance, CMJ, 505 COD, ∑skill, and Stroop test interference were entered simultaneously as predictors. Playing position was included as a categorical covariate, with goalkeeper as the reference category. Standardized regression coefficients (β) with 95% CI were reported, and variance inflation factors (VIFs) were examined to assess multicollinearity. Receiver operating characteristic (ROC) curve analyses were performed to evaluate the discriminative ability of SoSCAT performance under DT conditions and completion time increase (ΔTime) for distinguishing between coach-rated talented and less talented players. The area under the curve (AUC), 95% CI, optimal cut-off values, sensitivity, and specificity were calculated. Statistical significance was set at *p* < 0.05.

## 3. Results

Table 1 presents the anthropometric and sociodemographic characteristics of the participants according to age category (U14, U16, and U18) and coach-rated talent status. Across age categories, significant differences were observed for all variables (*p* < 0.001), except for the number of goals scored during league competition (*p* = 0.267). Compared with less talented players, more talented players were significantly older (*p* = 0.003), accumulated more years of federated playing experience (*p* = 0.002), and scored a greater number of goals during the league season (*p* < 0.001), whereas no significant differences were observed in anthropometric characteristics.

Across age categories, older players generally demonstrated superior physical, technical, cognitive, and football-specific DT performance than younger players (Table 2). In particular, U18 players completed the SoSCAT in significantly less time under both single-task and DT conditions (both *p* < 0.001) and committed fewer decision errors than U14 and U16 players (*p* < 0.001). However, the increase in completion time induced by the DT condition did not differ significantly between age groups (*p* = 0.138; partial η^2^ = 0.022, 95% CI: 0.001–0.091). Similarly, DTC did not differ significantly between age categories (*p* = 0.868; ε^2^ ≈ 0.000, 95% CI: 0.000–0.036). Position-specific comparisons are presented in Supplementary Table S1.

Compared with less talented players, coach-rated talented players consistently demonstrated superior performance in the football-specific DT measures evaluated by the SoSCAT (Table 3). They completed the SoSCAT in significantly less time under both single-task (*p* = 0.002) and DT conditions (*p* < 0.001), showed a smaller increase in completion time under DT conditions (ΔTime; *p* < 0.001), exhibited a lower DTC (26.89% vs. 20.05%; *p* < 0.001; *r*rb = −0.351, 95% CI: −0.522 to −0.170), and committed fewer decision errors (*p* = 0.026). The largest adjusted effect among the SoSCAT-related comparisons was observed for performance under DT conditions (partial η^2^ = 0.190, 95% CI: 0.108–0.291). Talented players also achieved better performance in football-specific technical tests, including dribbling (*p* = 0.005), passing (*p* < 0.001), and the combined technical score (∑skill; *p* < 0.001). In addition, they showed superior physical performance, reflected by faster sprint times (*p* = 0.014), greater CMJ height (*p* < 0.001), and better 505 COD performance (*p* < 0.001), together with higher scores in the Wom-Rest (*p* < 0.001) and Vismem-Plan (*p* < 0.001) cognitive tests. No significant differences were observed for the 505 COD deficit (*p* = 0.580), Wom-Rest completion time (*p* = 0.107), or Stroop interference score (*p* = 0.866).

Table 4 presents the significant Pearson correlation coefficients between SoSCAT performance and the main demographic, technical, cognitive, and physical variables. Better SoSCAT performance under both single-task and DT conditions was significantly associated with older age, superior technical performance, better cognitive performance, and better physical performance (all *p* < 0.001). The strongest correlation was observed between SoSCAT performance under DT conditions and the combined technical score (∑skill; r = 0.605, 95% CI: 0.504–0.690), followed by the passing test (r = 0.516, 95% CI: 0.401–0.616), Vismem-Plan (r = −0.498, 95% CI: −0.615 to −0.360), Wom-Rest (r = −0.468, 95% CI: −0.590 to −0.325), and the number of league goals scored (r = −0.420, 95% CI: −0.533 to −0.292).

Multivariable linear regression analysis was performed to examine factors independently associated with SoSCAT completion time under DT conditions (Table 5). The overall model was significant (F (13,167) = 14.82, *p* < 0.001), explaining 53.6% of the variance in SoSCAT DT performance (R^2^ = 0.536; adjusted R^2^ = 0.500). After simultaneous adjustment for the other variables included in the model, age (β = −0.216, 95% CI: −0.335 to −0.097; *p* < 0.001), coach-rated talent status (β = −0.202, 95% CI: −0.322 to −0.082; *p* = 0.001), number of league goals (β = −0.151, 95% CI: −0.295 to −0.008; *p* = 0.039), and ∑skill (β = 0.369, 95% CI: 0.230–0.508; *p* < 0.001) remained significantly associated with SoSCAT DT completion time. No significant independent associations were observed for 10 m sprint performance, CMJ, 505 COD, or Stroop test interference (all *p* > 0.05).

**Figure 2:**
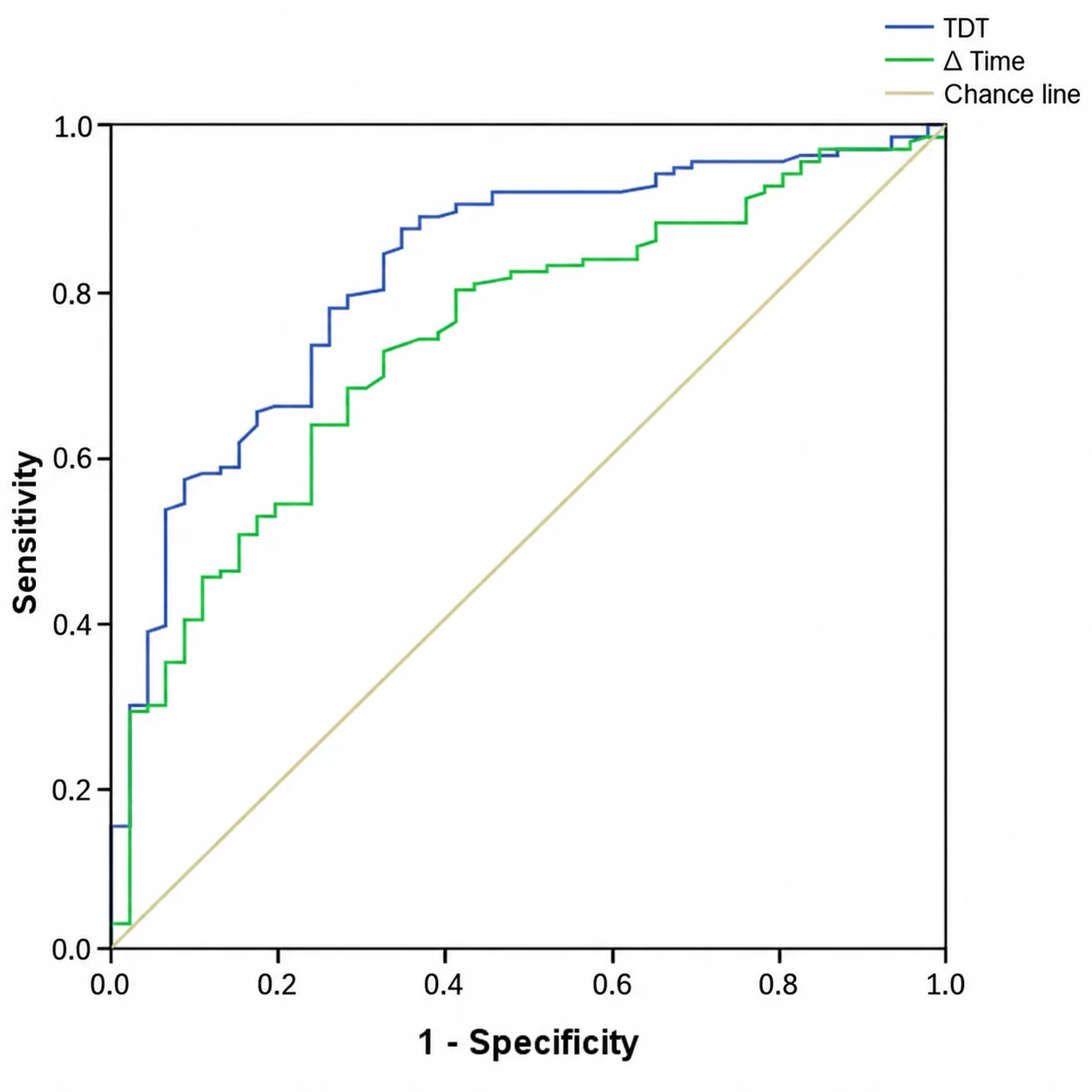
Receiver operating characteristic (ROC) curves illustrating the ability of SoSCAT performance under dual-task (DT) conditions and completion time increase (ΔTime) to discriminate between coach-rated talent groups.

Finally, ROC curve analyses demonstrated the discriminative ability of SoSCAT performance under DT conditions and completion time increase (ΔTime) for distinguishing between coach-rated talented and less talented players (Figure 2). The area under the curve (AUC) for SoSCAT under DT conditions was 0.822 (95% CI: 0.754–0.891; *p* < 0.001), with an optimal cut-off value of 41.28 s, corresponding to a sensitivity of 0.785 and a specificity of 0.717. For ΔTime, the AUC was 0.740 (95% CI: 0.661–0.819; *p* < 0.001), with an optimal cut-off value of 7.10 s, yielding a sensitivity of 0.711 and a specificity of 0.674.

## 4. Discussion

The present study demonstrated that football-specific performance under DT conditions, assessed using the SoSCAT, successfully discriminated between coach-rated talented and less talented youth football players. Specifically, players classified as more talented completed the SoSCAT faster under DT conditions, showed a smaller increase in completion time (ΔTime), exhibited a lower DTC, and committed fewer decision errors than their less talented counterparts. In addition, older players consistently achieved superior absolute performance under both single-task and DT conditions, suggesting age-related differences in football-specific cognitive–motor performance throughout youth development. Overall, these findings support the hypothesis that the ability to maintain football-specific technical performance under cognitive interference represents an important characteristic associated with coach-rated talent status in youth football.

The ability of the SoSCAT to discriminate between players with different coach-rated talent levels may be explained by its design, which integrates football-specific technical actions with cognitive demands resembling those encountered during match play [3]. Unlike traditional isolated technical tests, the SoSCAT requires players to continuously perceive visual information, select relevant stimuli, make rapid decisions, and execute precise technical actions under time pressure [34]. This integration of perceptual, cognitive, and motor demands may help explain why the test differentiated between coach-rated talent groups and is consistent with previous studies showing that players with superior technical and cognitive abilities experience smaller performance decrements when simultaneously managing motor and cognitive demands [29,33,45]. Importantly, the lower DTC observed in more talented players suggests that their superior performance was not limited to faster absolute completion times. Rather, these players experienced a smaller proportional deterioration when the cognitive demand was introduced. This finding may indicate a greater ability to preserve football-specific motor performance while simultaneously processing additional cognitive information, which represents a particularly relevant characteristic in the context of DT performance [2,3].

Beyond talent level, age also emerged as an important factor associated with superior performance under DT conditions. Older players not only completed the SoSCAT more rapidly but also committed fewer decision errors under DT conditions, indicating better absolute cognitive–motor performance across age categories [30]. However, neither ΔTime nor DTC differed significantly between age groups, suggesting that, despite clear age-related improvements in absolute SoSCAT performance, the additional interference induced by the cognitive demand remained relatively stable across developmental categories. Thus, superior performance in older players should not be interpreted as evidence of a systematically lower interference effect with increasing age. This developmental pattern is likely to reflect the combined influence of biological maturation, accumulated football experience, and repeated exposure to increasingly demanding competitive environments [28,29]. Biological maturation may be particularly relevant when interpreting these age- and coach-rated talent-related differences, as maturation during adolescence can influence anthropometric characteristics and physical performance and may also affect coaches’ perceptions of player quality. Recent evidence in youth football has shown that biological age is associated with coach-rated player quality, suggesting that more biologically mature players may be perceived as higher-quality performers, partly because of maturation-related physical advantages [46]. As biological maturation was not assessed in the present study, its potential contribution to the differences observed between age categories and coach-rated talent groups cannot be determined.

One possible explanation for the age and talent-related differences observed in the present study lies in the development of perceptual-cognitive expertise, particularly visual search behaviour and anticipation skills. Evidence indicates that higher-level football players adopt more efficient visual search strategies, directing their gaze towards task-relevant sources of information while reducing attention to irrelevant stimuli or excessive monitoring of ball control [47,48,49]. Such perceptual behaviours facilitate earlier anticipation of opponents’ actions and more accurate selection of appropriate responses, thereby improving decision-making during play. These mechanisms may partly explain why talented and older players in the present study completed the SoSCAT more efficiently and committed fewer decision errors under DT conditions. In addition, previous studies have suggested that visual search strategies are closely associated with technical mastery, reinforcing the relationship between perceptual-cognitive expertise, motor learning, and football performance [31,39].

The significant associations observed between SoSCAT performance and technical, cognitive, and physical variables reinforce the multidimensional nature of football performance [4,5]. Rather than reflecting a single technical ability, successful performance in the SoSCAT appears to depend on the integration of several complementary capacities relevant to football performance. The strongest bivariate relationship was observed with football-specific technical performance, particularly the combined technical score (∑skill), highlighting the central role of technical proficiency when executing complex actions under DT conditions. Significant associations with cognitive measures, including working memory, spatial perception, processing speed and planning ability, and with physical measures such as sprinting, change-of-direction performance, and CMJ, further indicate that SoSCAT performance is related to multiple performance domains. However, these bivariate relationships should be distinguished from the findings of the multivariable analysis. When relevant predictors were considered simultaneously, only age, coach-rated talent status, number of league goals, and ∑skill remained independently associated with SoSCAT completion time under DT conditions. In particular, ∑skill showed the strongest independent association (β = 0.369), whereas sprint performance, CMJ, 505 COD, and Stroop interference were no longer independently associated with SoSCAT DT performance. These findings suggest that some of the physical and cognitive associations observed at the bivariate level may reflect shared variance with age, technical performance, or other player characteristics. Overall, the multivariable model explained 53.6% of the variance in SoSCAT DT performance, supporting the view that football-specific DT performance is multidimensional [4], while also highlighting the particular contribution of football-specific technical proficiency.

The ROC analysis provides additional support for the potential usefulness of the SoSCAT within multidimensional player assessment frameworks in youth football. In particular, the AUC obtained under DT conditions (0.822) indicates good discriminative ability for distinguishing between players classified by their coaches as more or less talented. Although this value should not be interpreted as evidence that the SoSCAT can independently identify football talent, it suggests that football-specific DT performance captures characteristics associated with coach-rated talent status. This interpretation is further supported by the multivariable analysis, in which coach-rated talent status remained independently associated with SoSCAT DT completion time after simultaneous consideration of age, technical and physical performance, league goals, Stroop test interference, and playing position. Together, these findings support the potential value of SoSCAT performance as an objective component of multidimensional player assessment.

From an applied perspective, the present findings suggest that the SoSCAT may represent a valuable complementary tool for multidimensional player assessment and development in youth football. Rather than replacing traditional assessments or coaches’ evaluations, the SoSCAT provides objective information regarding players’ ability to integrate technical execution and cognitive processing under football-specific DT conditions. Consequently, the test may complement conventional physical, technical, and cognitive assessments, contributing to a more holistic evaluation of player performance [4]. In addition, because the SoSCAT provides objective measures such as completion time under DT conditions, completion time increase (ΔTime), decision errors, and DTC, future longitudinal applications could examine its usefulness for monitoring players’ cognitive–motor development throughout the competitive season [50]. Periodic assessments (e.g., pre-season, mid-season, and end-season) could help determine whether players become more efficient at maintaining football-specific performance under DT conditions, as reflected by reductions in ΔTime, DTC, and decision errors over time. Such information may assist coaches in evaluating individual player development, monitoring the effectiveness of training programmes, and supporting evidence-based decision-making throughout the talent development process.

### Limitations and Strengths

This study has several limitations that should be acknowledged. First, the cross-sectional design precludes establishing causal relationships between the variables analysed. Consequently, longitudinal studies are required to determine how football-specific DT performance evolves throughout player development and whether changes in SoSCAT performance are associated with future sporting achievement. Second, the sample consisted exclusively of male youth football players, limiting the generalisability of the findings to female football. Future research should therefore include female players to determine whether similar relationships exist across sexes. Third, biological maturation was not assessed in the present study. This represents a relevant limitation given the wide age range of the sample (12–18 years) and the substantial inter-individual variability in maturation that occurs during adolescence. Maturation-related differences may influence anthropometric and physical performance and may also affect coaches’ perceptions of player quality. Therefore, biological maturation may have contributed, at least partly, to some of the differences observed between age categories and coach-rated talent groups. Future studies should incorporate biological maturation indicators, such as maturity offset or estimated age at peak height velocity (APHV), to better distinguish the influence of chronological age, maturation, and accumulated football experience on football-specific DT performance [46]. Fourth, talent classification was based on coaches’ evaluations rather than on an objective external criterion. Although all participating coaches held official coaching qualifications, regularly observed players during training and competition, and received the same instructions for the classification procedure, no predefined multidimensional rating criteria or standardized assessment scale were used. Furthermore, because each player was evaluated exclusively by his respective head coach, inter-rater reliability could not be determined. Therefore, the coach-rated classification should be interpreted as an expert-based assessment of players’ overall performance rather than as an objective measure of talent. Future studies should combine expert evaluations with standardized multidimensional rating procedures, multiple independent evaluators, objective performance indicators, and longitudinal follow-up to determine whether football-specific DT performance contributes to prospective talent identification and the prediction of future competitive achievement. Finally, although the SoSCAT demonstrated promising discriminative ability, it was not directly compared with other football-specific DT assessments, such as the Stroop Task Football Test (STFT) or Loughborough Soccer Passing Test (LSPT) [29,51]. Future studies should therefore compare the measurement properties and practical utility of different football-specific DT protocols.

Despite these limitations, the present study also has several important strengths. The SoSCAT was administered on a football pitch, integrating football-specific technical execution with perceptual-cognitive demands within a representative field-based setting. Furthermore, the study included a relatively large sample of youth football players spanning multiple developmental categories, enabling the analysis of age and talent-related differences. Finally, the combination of group comparisons, correlation analyses, multivariable regression analysis, ROC analysis, and football-specific performance measures provides a comprehensive evaluation of the factors associated with DT performance in youth football.

## 5. Conclusions

In conclusion, football-specific DT performance demonstrated good discriminative ability for distinguishing between coach-rated talented and less talented youth football players. Players classified as more talented demonstrated superior performance under DT conditions, characterised by faster completion times, a smaller increase in completion time (ΔTime), lower DTC, and fewer decision errors. Furthermore, age, coach-rated talent status, number of league goals, and football-specific technical performance (∑skill) remained independently associated with SoSCAT performance under DT conditions in the multivariable analysis. These findings reinforce the multidimensional nature of football performance, highlighting the potential value of integrating complementary performance domains during talent assessment. Owing to its ecological validity and its ability to simultaneously assess football-specific technical execution and cognitive processing, the SoSCAT may represent a valuable complementary tool for multidimensional player assessment and longitudinal monitoring within youth football development programmes.

## Figures and Tables

**Table 1:** Anthropometric and sociodemographic characteristics of football players according to age category and coach-rated talent status.

	Category					Coach-Rated Talent Status			
	U14 n = 70	U16 n = 63	U18 n = 48	*p*-Value	η^2^ (95% CI)	Less Talented n = 135	More Talented n = 46	*p*-Value	Hedges’ g (95% CI)
Age (Years)	12.94 (0.74)	15.00 (0.70)	17.06 (0.81)	<0.001	0.832 (0.791–0.862)	14.53 (1.82)	15.39 (1.61)	0.003	0.48 (0.15–0.82)
Weight (kg)	50.34 (11.16)	62.22 (7.66)	68.56 (8.31)	<0.001	0.399 (0.291–0.490)	58.70 (11.95)	61.10 (11.95)	0.176	0.20 (−0.13–0.53)
Height (m)	1.62 (0.10)	1.73 (0.08)	1.74 (0.06)	<0.001	0.310 (0.201–0.407)	1.69 (0.10)	1.71 (0.09)	0.135	0.29 (−0.05–0.62)
BMI (kg/m^2^)	18.84 (2.40)	20.69 (2.02)	22.59 (2.31)	<0.001	0.310 (0.201–0.407)	20.43 (2.59)	20.64 (2.99)	0.648	0.08 (−0.26–0.41)
Nº years playing football (federated)	4.23 (2.21)	6.49 (2.56)	7.44 (3.07)	<0.001	0.218 (0.117–0.316)	5.50 (2.91)	6.93 (2.63)	0.002	0.50 (0.16–0.84)
Nº of goals in the league matches	3.03 (4.35)	3.89 (4.96)	4.56 (5.93)	0.267	0.015 (0.000–0.061)	2.50 (3.35)	7.35 (7.07)	<0.001	1.05 (0.70–1.40)

Abbreviations: U14, U16, U18: Under-14 years, Under-16 years, and Under-18 years, respectively; BMI: Body mass index. Data are expressed as mean (standard deviation). Effect sizes are reported as eta squared (η^2^) with 95% confidence intervals (CIs) for comparisons between age categories and Hedges’ *g* with 95% CIs for comparisons between coach-rated talent groups.

**Table 2:** Performance in physical, technical, cognitive, and football-specific dual-task (DT) measures according to age category.

Category					
	U14 n = 70	U16 n = 63	U18 n = 48	*p*-Value	Effect Size (95% CI)
10-metre sprint test (s)	2.08 (0.11)	2.01 (0.17)	1.92 (0.14)	<0.001	η^2^ = 0.171 (0.091–0.299)
CMJ (cm) #	29.99 (4.09)	31.78 (5.05)	35.23 (4.73)	<0.001	ε^2^ = 0.153 (0.074–0.265)
505 COD (mean of right and left turns) (s)	2.56 (0.18)	2.47 (0.13)	2.45 (0.20)	<0.001	η^2^ = 0.082 (0.025–0.185)
505 COD (deficit in COD) #	1.23 (0.07)	1.23 (0.09)	1.28 (0.09)	0.013	ε^2^ = 0.037 (0.000–0.130)
Football dribbling test (s)	32.13 (3.29)	29.12 (3.51)	28.48 (3.27)	<0.001	η^2^ = 0.191 (0.102–0.309)
Football passing test (s)	49.57 (5.42)	48.20 (5.20)	45.32 (5.12)	<0.001	η^2^ = 0.095 (0.036–0.196)
∑skill (=passing + dribbling)	81.70 (7.05)	77.31 (6.61)	73.79 (7.72)	<0.001	η^2^ = 0.170 (0.083–0.294)
Wom-Rest (%) #	74.42 (4.76)	77.35 (4.28)	81.86 (5.78)	<0.001	ε^2^ = 0.222 (0.109–0.383)
Wom-Rest (s)	2.37 (0.30)	2.08 (0.37)	1.87 (0.30)	<0.001	η^2^ = 0.288 (0.190–0.409)
Vismem-Plan (%) #	41.58 (6.25)	51.96 (6.08)	59.86 (7.35)	<0.001	ε^2^ = 0.563 (0.458–0.665)
Stroop test interference	−1.69 (11.57)	4.92 (10.49)	1.50 (9.27)	0.002	η^2^ = 0.067 (0.018–0.162)
SoSCAT (single-task) (s)	38.05 (5.45)	34.24 (4.04)	33.89 (3.42)	<0.001	η^2^ = 0.158 (0.076–0.269)
SoSCAT (dual-task) (s) ≠	47.57 (5.25)	42.41 (5.36)	42.08 (4.77)	<0.001	partial η^2^ = 0.167 (0.089–0.276)
ΔTime completion time increase (s) ≠	9.52 (5.07)	8.16 (3.22)	8.19 (3.22)	0.138	partial η^2^ = 0.022 (0.001–0.091)
DTC (%) #	26.64 (17.68)	24.07 (9.58)	24.40 (9.81)	0.868	ε^2^ ≈ 0.000 (0.000–0.036)
Decision errors #	2.23 (1.23)	1.67 (1.14)	1.35 (0.91)	<0.001	ε^2^ = 0.092 (0.030–0.192)

**Abbreviations:** U14, U16, U18: Under-14, Under-16, and Under-18, respectively; CMJ: Countermovement Jump; 505 COD: 505 Change-of-Direction Test; Wom-Rest and Vismem-Plan: cognitive assessment tests; SoSCAT: Soccer Skills and Cognitive Aptitude Test; DTC: Dual-Task Cost. **Notes:** Data are expressed as mean (standard deviation). ≠: adjusted for decision errors; #: analysed using non-parametric methods. Effect sizes are reported as eta squared (η^2^), epsilon squared (ε^2^), or partial eta squared (partial η^2^), as appropriate, with 95% confidence intervals (CIs). Higher values indicate better performance for Wom-Rest (%) and Vismem-Plan (%), whereas lower values indicate better performance for all other variables.

**Table 3:** Performance in physical, technical, cognitive, and football-specific dual-task (DT) measures according to coach-rated talent status.

Coach-Rated Talent Status				
	Less Talented n = 135	More Talented n = 46	*p*-Value	Effect Size (95% CI)
10-metre sprint test (s) *	2.04 (0.16)	1.95 (0.13)	0.014	partial η^2^ = 0.034 (0.002–0.094)
CMJ (cm) #	31.02 (4.50)	34.88 (5.47)	<0.001	r_r__β_ = 0.389 (0.207–0.569)
505 COD (mean of right and left turns) (s)	2.53 (0.18)	2.41 (0.13)	<0.001	Hedges’ g = −0.694 (−0.992 to −0.436)
505 COD (deficit in COD) #	1.25 (0.09)	1.24 (0.08)	0.580	r_r__β_ = −0.055 (−0.248–0.135)
Football dribbling test (s) *	30.71 (3.62)	28.37 (3.49)	0.005	partial η^2^ = 0.044 (0.004–0.119)
Football passing test (s) *	48.87 (5.28)	45.33 (5.37)	<0.001	partial η^2^ = 0.065 (0.013–0.154)
∑skill (=passing + dribbling) *	79.57 (7.47)	73.69 (6.86)	<0.001	partial η^2^ = 0.081 (0.024–0.167)
Wom-Rest (%) #	76.22 (4.70)	80.33 (6.77)	<0.001	r_r__β_ = 0.408 (0.190–0.621)
Wom-Rest (s) *	2.20 (0.37)	2.00 (0.39)	0.107	partial η^2^ = 0.019 (0.000–0.096)
Vismem- Plan (%) #	48.24 (9.21)	54.50 (9.89)	<0.001	r_r__β_ = 0.347 (0.137–0.541)
Stroop test interference *	1.12 (11.39)	2.45 (9.53)	0.866	partial η^2^ = <0.001 (0.000–0.031)
SoSCAT (single-task) (s) *	36.41 (5.05)	33.29 (3.44)	0.002	partial η^2^ = 0.052 (0.012–0.115)
SoSCAT (dual-task) (s) ≠	45.85 (5.47)	39.81 (3.94)	<0.001	partial η^2^ = 0.190 (0.108–0.291)
ΔTime completion time increase (s) ≠	9.44 (4.10)	6.52 (3.10)	<0.001	partial η^2^ = 0.095 (0.030–0.180)
DTC (%) #	26.89 (13.69)	20.05 (10.55)	<0.001	r_rb = −0.351 (−0.522 to −0.170)
Decision errors #	1.92 (1.20)	1.46 (1.00)	0.026	r_r__β_ = −0.211 (−0.381 to −0.044)

**Abbreviations:** CMJ: Countermovement Jump; 505 COD: 505 Change-of-Direction Test; Wom-Rest and Vismem-Plan: cognitive assessment tests; SoSCAT: Soccer Skills and Cognitive Aptitude Test; DTC: Dual-Task Cost. **Notes:** Data are expressed as mean (standard deviation). ≠: adjusted for decision errors; #: analysed using non-parametric methods; *: adjusted for age. Effect sizes are reported as partial eta squared (partial η^2^) for adjusted comparisons, Hedges’ *g* for parametric comparisons, and rank-biserial correlation (*r*rb) for non-parametric comparisons, with 95% confidence intervals (CIs). Higher values indicate better performance for Wom-Rest (%) and Vismem-Plan (%), whereas lower values indicate better performance for all other variables.

**Table 4:** Pearson correlation coefficients between SoSCAT performance and demographic, technical, cognitive, and physical variables.

Variables	r	95% CI	*p*-Value
**Demographic variables**			
Age vs. SoSCAT (single-task)	−0.336	−0.460 to −0.200	<0.001
Age vs. SoSCAT (dual-task)	−0.396	−0.512 to −0.266	<0.001
Age vs. Decision errors	−0.310	−0.436 to −0.172	<0.001
**Technical** **performance**			
SoSCAT (single-task) vs. Number of league goals	−0.297	−0.424 to −0.158	<0.001
SoSCAT (dual-task) vs. Number of league goals	−0.420	−0.533 to −0.292	<0.001
SoSCAT (single-task) vs. Football passing test	0.429	0.302 to 0.541	<0.001
SoSCAT (dual-task) vs. Football passing skills test	0.516	0.401 to 0.616	<0.001
SoSCAT (dual-task) vs. Football dribbling test	0.494	0.376 to 0.597	<0.001
SoSCAT (single-task) vs. ∑skill (=passing + dribbling)	0.452	0.327 to 0.561	<0.001
SoSCAT (dual-task) vs. ∑skill (=passing + dribbling)	0.605	0.504 to 0.690	<0.001
Decision errors vs. ∑skill (=passing + dribbling)	0.268	0.127 to 0.398	<0.001
**Cognitive performance**			
SoSCAT (single-task) vs. Wom-Rest	−0.425	−0.554 to −0.276	<0.001
SoSCAT (dual-task) vs. Wom-Rest	−0.468	−0.590 to −0.325	<0.001
SoSCAT (single-task) vs. Vismem-Plan	−0.371	−0.508 to −0.217	<0.001
SoSCAT (dual-task) vs. Vismem-Plan	−0.498	−0.615 to −0.360	<0.001
**Physical performance**			
SoSCAT (dual-task) vs. 505 COD	0.408	0.278 to 0.522	<0.001
SoSCAT (dual-task) vs. 10-metre sprint test	0.356	0.221 to 0.477	<0.001
SoSCAT (dual-task) vs. CMJ	−0.376	−0.495 to −0.243	<0.001
Δ Time completion time increase vs. 505 COD	0.390	0.259 to 0.507	<0.001
Δ Time completion time increase vs. Football dribbling test	0.335	0.199 to 0.458	<0.001
Δ Time completion time increase vs. ∑skill (=passing + dribbling)	0.315	0.177 to 0.440	<0.001
DTC vs. 505 COD	0.354	0.220 to 0.475	<0.001

Abbreviations: CMJ: Countermovement Jump; 505 COD: 505 Change-of-Direction Test; Wom-Rest and Vismem-Plan: cognitive assessment tests; SoSCAT: Soccer Skills and Cognitive Aptitude Test; ΔTime: completion time increase; DTC: Dual-Task Cost. Note: Pearson correlation coefficients (*r*) are reported with 95% confidence intervals (CIs). Analyses involving Wom-Rest and Vismem-Plan were based on available cases (n = 136).

**Table 5:** Multivariable linear regression analysis of factors associated with football-specific dual-task (DT) performance.

	Standardized β	95% CI	*p*-Value	VIF
Age	−0.216	−0.335 to −0.097	<0.001	1.30
Coach-rated talent status	−0.202	−0.322 to −0.082	0.001	1.33
Nº of goals in the league matches	−0.151	−0.295 to −0.008	0.039	1.90
10-metre sprint test	−0.058	−0.204 to 0.088	0.433	1.97
CMJ	−0.033	−0.172 to 0.106	0.642	1.79
505 COD	0.066	−0.073 to 0.205	0.349	1.78
∑skill (=passing + dribbling)	0.369	0.230 to 0.508	<0.001	1.78
Stroop test interference	0.078	−0.031 to 0.188	0.158	1.10

Abbreviations: SoSCAT: Soccer Skills and Cognitive Aptitude Test; CMJ: countermovement jump; 505 COD: 505 Change-of-Direction Test; VIF: variance inflation factor; CI: confidence interval. Notes: SoSCAT completion time under dual-task conditions was entered as the dependent variable. All predictors were entered simultaneously. Game position was included as a categorical covariate, with goalkeeper as the reference category. Standardized regression coefficients (β) are presented with 95% confidence intervals. Lower SoSCAT completion times indicate better performance. Model statistics: N = 181; R^2^ = 0.536; adjusted R^2^ = 0.500; F (13,167) = 14.82; *p* < 0.001.

## Data Availability

The data that support the findings of this study are available from the corresponding author upon reasonable request.

## Supplementary Materials

The following supporting information can be downloaded at https://www.mdpi.com/article/10.3390/sports14090399/s1, Supplementary Table S1: Performance in physical, technical, cognitive, and football-specific dual-task measures according to playing position.

## Abbreviations

The following abbreviations are used in this manuscript:

DT	Dual-Task
DTC	Dual-Task Cost
SoSCAT	Soccer Skills and Cognitive Aptitude Test
CMJ	Countermovement Jump
505 COD	505 Change-of-Direction Test
STFT	Stroop Task Football Test
LSPT	Loughborough Soccer Passing Test
