# Supplementary material for: Thinking While Playing: Football-Specific Dual-Task Performance and Coach-Rated Talent in Youth Football
Source: Sports (Basel). 2026 Sep 10;14(9):399. doi: 10.3390/sports14090399 (PMC13611753; doi:10.3390/sports14090399)
Supplement: Supplementary file 1 [file sports-14-00399-s001.zip › sports-4488225-supplementary.pdf]

Supplementary Table S1. Performance in physical, technical, cognitive, and football-specific dual-task measures according to playing position.

	Positions							
	**Keeper n=19**	**Centre-back n=27**	**Right/left back n=37**	**Midfielder n=41**	**Winger n=33**	**Forward n=24**	**p-value**	**Effect size**
10-metre sprint test (s)	2.05 (0.17)	1.97 (0.14)	2.03 (0.18)	1.99 (0.13)	2.03 (0.14)	2.03 (0.17)	0.400	$\eta^2 = 0.029$
CMJ (cm) #	31.61 (4.96)	32.21 (4.98)	31.74 (4.76)	31.36 (4.90)	33.04 (5.37)	32.15 (5.62)	0.755	$\varepsilon^2 \approx 0.000$*
505 COD (mean of right and left turns) (s)	2.59 (0.21)	2.47 (0.14)	2.52 (0.17)	2.48 (0.13)	2.45 (0.12)	2.53 (0.29)	**0.058**	$\eta^2 = 0.059$
505 COD (deficit in COD) #	1.27 (0.11)	1.26 (0.09)	1.25 (0.08)	1.25 (0.08)	1.21 (0.07)	1.25 (0.10)	0.074	$\varepsilon^2 = 0.029$
Football dribbling test (s)	31.73 (2.98)	29.77 (3.84)	29.79 (3.37)	29.18 (3.70)	30.46 (4.12)	30.82 (3.82)	0.165	$\eta^2 = 0.044$
Football passing test (s)	51.44 (4.78)	47.00 (6.39)	47.70 (5.04)	47.04 (5.86)	48.64 (5.38)	47.36 (4.46)	0.061	$\eta^2 = 0.058$
∑skill (=passing + dribbling)	83.17 (6.20)	76.77 (8.61)	77.49 (6.86)	76.22 (7.83)	79.11 (7.93)	78.18 (7.43)	**0.030**	$\eta^2 = 0.068$
Wom-Rest (%) #	74.92 (4.77)	78.40 (5.28)	76.83 (4.54)	78.36 (5.55)	75.30 (6.14)	80.11 (6.85)	0.055	$\varepsilon^2 = 0.045$
Wom-Rest (s)	2.19 (0.46)	2.05 (0.39)	2.24 (0.33)	2.08 (0.35)	2.24 (0.37)	2.00 (0.41)	0.160	$\eta^2 = 0.059$
Vismem-Plan (%) #	51.62 (12.99)	52.70 (8.74)	47.00 (8.10)	51.52 (9.89)	46.57 (8.43)	52.89 (10.98)	0.111	$\varepsilon^2 = 0.030$
Stroop test interference	0.72 (13.69)	2.79 (8.80)	1.89 (9.19)	3.10 (10.91)	1.18 (13.66)	−2.55 (8.91)	0.456	$\eta^2 = 0.026$
SoSCAT (single-task) (s)	39.52 (5.18)	35.78 (4.14)	34.87 (4.41)	34.23 (4.37)	35.81 (5.37)	35.62 (5.02)	**0.005**	$\eta^2 = 0.091$
SoSCAT (dual-task) (s) ≠	49.69 (5.61)	44.20 (4.53)	44.11 (5.09)	42.96 (6.55)	43.84 (5.18)	43.48 (5.56)	**0.003**	partial $\eta^2 = 0.098$
ΔTime completion time increase (s) ≠	10.17 (4.42)	8.41 (2.40)	9.24 (4.74)	8.73 (4.59)	8.03 (3.16)	7.86 (4.28)	0.430	partial $\eta^2 = 0.027$
Decision errors #	2.37 (1.42)	1.48 (0.85)	1.54 (1.22)	1.90 (1.22)	1.79 (1.05)	1.96(1.16)	0.118	$\varepsilon^2 = 0.022$

Abbreviations: CMJ: Countermovement Jump; 505 COD: 505 Change-of-Direction Test; Wom-Rest and Vismem-Plan: cognitive assessment tests; SoSCAT: Soccer Skills and Cognitive Aptitude Test; ΔTime: completion time increase. **Notes:** Data are expressed as mean (standard deviation). ≠: adjusted for decision errors; #: analysed using non-parametric methods. Effect sizes are reported as eta squared (η^2) for parametric comparisons, epsilon squared (ε^2) for non-parametric comparisons, and partial eta squared (partial η^2) for adjusted comparisons. Analyses involving Wom-Rest and Vismem-Plan were based on the available subsample (n = 136). Higher values indicate better performance for Wom-Rest (%) and Vismem-Plan (%), whereas lower values indicate better performance for all other variables. A slightly negative ε^2 estimate was obtained for CMJ and was reported as 0.000

.
